# Nonclassical Philadelphia-positive FISH signal patterns in CML and seven cases with variant Philadelphia

**DOI:** 10.55730/1300-0144.6171

**Published:** 2025-09-02

**Authors:** Sevgi IŞIK, Oğuz ÇİLİNGİR, Gülçin GÜNDEN, Filiz YAVAŞOĞLU, Hülya ÖZEN, Mert Burak RAŞAN, Beyhan DURAK ARAS

**Affiliations:** 1 Department of Medical Genetics, Faculty of Medicine, Eskişehir Osmangazi University, Eskisehir, Turkiye; 2 Department of Hematology, Faculty of Medicine, Eskişehir Osmangazi University, Eskisehir, Turkiye; 3 Department of Medical Informatics, Gülhane Faculty of Medicine, University of Health Sciences, Ankara, Turkiye

**Keywords:** Chronic myeloid leukemia, fluorescence in situ hybridization, variant t(9;22), conventional cytogenetics

## Abstract

**Background/aim:**

Chronic myeloid leukemia (CML) is characterized by the Philadelphia chromosome (Ph), which occurs as a result of t(9;22). In 5%–10% of CML cases, variant t(9;22)s are observed. Additionally, while t(9;22) is formed, deletions can be observed in chromosomes 9 and 22. These deletions are observed more frequently in variant t(9;22). There is conflicting information in the literature about the prognostic effects of variant t(9;22) and deletions of 9q and 22q. There is also limited information about the frequency and breakpoints of other chromosomes involved in the variant t(9;22). Signal patterns other than the classical signal pattern of FISH analysis indicate deletions and variant translocations. In this study, we aimed to investigate the clinical significance of nonclassical FISH signal patterns and to determine the frequency of variant translocations.

**Materials and methods:**

Bone marrow samples from 231 newly diagnosed CML patients were analyzed by conventional cytogenetics and FISH.

**Results:**

As a result of FISH analysis, nonclassical FISH signal patterns were detected in 49/231 cases, and variant t(9;22) was detected in seven cases by conventional cytogenetics. It was determined that chromosomes 1, 3, 5, 7, 8, and 21 were involved in variant t(9;22). When cases with classical and nonclassical signal patterns were compared in terms of 6th- and 12th-month treatment responses, survival times, and treatment changes, it was found that cases with classical signal patterns had significantly higher treatment responses at the 6th month (p < 0.001).

**Conclusion:**

Because variant translocations are extremely rare and involve many different chromosomal breaks, a large number of cases are needed to clearly understand their prognostic implications. Due to the limitations of conventional cytogenetic analyses, it should be considered in patient follow-up that nonclassical FISH signal patterns indicating deletions and/or variant translocations may cause a delay in obtaining a complete cytogenetic response at the 6th month.

## 1. Introduction

Chronic myeloid leukemia is characterized by a balanced translocation between chromosomes 9 and 22. As a result of this translocation, the Ph chromosome and the *BCR::ABL1* fusion gene are formed. The fusion gene has tyrosine kinase activity. The Philadelphia chromosome is detected in 95% of CML cases. However, 5%–10% of cases with t(9;22) are observed as variant translocations (vPh) that involve chromosomes 9, 22 and at least one additional chromosome [1]. Variant translocations can occur as a result of two different mechanisms: a one-step mechanism, in which a third or fourth chromosome is involved and translocated simultaneously, and a two-step mechanism, in which two translocations occur. The first translocation is the classic t(9;22), followed by translocations involving additional chromosomes [2].

There is no clear information in the literature about the prognostic impact of variant t(9;22). While some researchers argue that vPh causes clinical outcomes similar to those of classical t(9;22) [1–3], others state that it leads to a more aggressive disease course [4–6]. There are also very limited data in the literature about the frequency and breakpoints of the additional chromosomes involved in variant t(9;22). However, it is known that translocations can sometimes be cryptic and masked. In addition, conventional cytogenetics is not always successful, and the chromosome banding quality is not always adequate. In addition, *Abelson* (*ABL1*) and breakpoint cluster region (*BCR*) deletions can be observed in CML. Some of these deletions occur due to variant translocations, and their prognostic effects are also a matter of debate. All these situations should be considered when a nonclassical signal pattern is observed with the fluorescence in situ hybridization (FISH) test [7].

In our study, we aimed to investigate the clinical effects of nonclassical FISH-positive signal patterns by evaluating a group of 231 newly diagnosed CML patients and to determine the frequency of additional chromosomes involved in variant t(9;22).

## 2. Material and methods

The study involved 231 newly diagnosed CML patients in the Hematology Department between 2005 and 2023. Each individual provided a signed consent form.

The study was conducted according to the guidelines of the Declaration of Helsinki and was approved by the relevant Clinical Practice Ethics Committee (2021–49).

### 2.1. Treatment and follow-up of cases

Before starting the treatment, a hemogram and a biochemistry panel including total protein, albumin, creatinine, lactate dehydrogenase, glucose, uric acid, alkaline phosphatase, and total and direct bilirubin were studied in all patients. Bone marrow aspiration and biopsy were performed. All patients diagnosed with chronic myeloid leukemia received imatinib 400 mg/day. Periodic hemogram, biochemical tests, bone marrow aspiration, and cytogenetic examinations were performed in all patients receiving treatment.

Follow-up of the cases was evaluated in line with the European LeukemiaNet (ELN) recommendations [8].

### 2.2. Cytogenetic and FISH analysis

Bone marrow samples were harvested after short-term (direct, 24 and 48 h) culture. The classical G-banding protocol was applied. The results were reported according to the International System for Human Cytogenomic Nomenclature (ISCN 2020).

The FISH protocol was applied to directly cultured bone marrow preparations. The probes used were the Locus-Specific Identifier BCR/ABL Dual-Color, Dual-Fusion Translocation Probe (Abbott Molecular-Vysis, Des Plaines, IL, USA) and the ZytoLight SPEC BCR/ABL1 Dual Color Dual Fusion Probe (ZytoVision GmbH, Bremerhaven, Germany). These probes detect the *BCR::ABL1* fusion gene on the derivative chromosomes.

As a result of the analysis of a normal cell using these probes, a pattern of two orange signals (two normal chromosome 9) and two green signals (two normal chromosome 22) is observed. In classical t(9;22), the expected signal pattern is 2F1O1G (two fusion signals, one orange, and one green). In a cell with a variant translocation, the signal pattern varies.

At least 200 cells were evaluated in FISH analysis, and the results were reported according to ISCN 2020.

Complete cytogenetic response (CCyR) was evaluated according to FISH test results at 6 months after diagnosis.

### 2.3. Molecular analysis

The p210, p230, and p190 transcripts of the *BCR::ABL1* fusion gene were analyzed by real-time reverse transcription polymerase chain reaction (RT-PCR) using the manufacturer’s suggested protocol [geneMAP BCR-ABL1 p210 (Mbcr) Detection Kit (BCR210-RT48), geneMAP BCR-ABL1 p190 (mbcr) Detection Kit (BCR190-RT48), and geneMAP BCR-ABL1 p230 (μbcr) Detection Kit (BCR230-RT48); GenMark Diagnostics, Carlsbad, CA, USA].

Major molecular response (MMR) was evaluated according to RT-PCR test results at 12 months after diagnosis.

### 2.4. Statistical analysis

Data analysis was performed with SPSS version 21.0 (IBM Corp., Armonk, NY, USA). Descriptive statistics of quantitative variables were shown as mean ± standard deviation and median (Q1–Q3), while qualitative variables were presented as counts and percentages. Normality of quantitative variables was evaluated with the Shapiro–Wilk test. The Mann–Whitney U test was used for comparisons between two nonnormally distributed groups. McNemar’s test was conducted to assess differences in dichotomous dependent variables between two related methods. The relationships between qualitative variables were assessed with the chi square test. The log-rank test was used to compare two survival functions. Statistical significance was defined as p < 0.05.

## 3. Results

A total of 231 newly diagnosed CML patients in our faculty were included in the study. As a result of FISH analysis, the classical signal pattern was detected in 182 cases. The signal patterns detected in the remaining 49 cases are shown in Supplementary Table.

Of the 49 cases with vPh, 28 were male and 21 were female, and the mean age was 71.33 ± 20.23.

The conventional cytogenetics test was successful in 22 of 49 cases, and a rare variant t(9;22) was detected in seven of them (cases #1–7). Of the variant t(9;22) cases, three occurred as a result of a one-step mechanism, and three occurred as a result of a two-step mechanism. It was observed that three of them were three-way and three were four-way translocations. Tyrosine kinase inhibitor (TKI) mutation information could be obtained for three of the seven cases with variant Ph, and the mutation was negative in these three patients (cases #2, #5, and #6).

The demographic and clinical information of the cases are listed in Table 1. It should be noted that differences in total numbers across variables in Table are due to missing clinical data for some patients.

A total of 182 patients with the classical Ph chromosome detected by FISH were identified. However, 82 cases in which the Ph chromosome was detected as isolated by conventional cytogenetic analysis were included in the case group. Cases with classical signal patterns in which chromosomes could not be obtained, and cases with additional cytogenetic abnormalities, were excluded to more clearly evaluate the prognostic impact of the nonclassical FISH pattern. Since 73 of 82 cases could be followed up, 73 cases were included in the statistical analyses. Of the 73 cases, 38 were male and 35 were female. The mean age was 61.41 ± 15.42.

It was determined that 28 of 49 cases with a nonclassical FISH pattern were switched to second-generation drugs in their treatment. Second-generation drugs were used in 28 of 72 cases in which clinical findings were obtained and the classical Ph was detected.

When cases with nonclassical and classical Ph chromosomes were compared in terms of age, sex, switch to second-generation drug use, 12th-month treatment response, survival, and life expectancy, no difference was found between the two groups (p > 0.05). However, when compared in terms of 6th-month CCyR, it was found that the cases with the classical Ph chromosome had a statistically significantly higher rate (p < 0.001).

When the 6th- and 12th-month treatment responses of cases with a nonclassical FISH signal pattern were compared, it was found that the MMR rate at the 12th-month was significantly higher than the 6th-month CCyR rate (p = 0.013). Similarly, in cases with classical Ph, although six patients had achieved CCyR at 6 months, they did not reach MMR at 12 months. This finding suggests that, within the classical Ph group, the early cytogenetic response rate was statistically higher than the molecular response rate (p = 0.031); however, this did not translate into a sustained molecular response over time.

## 4. Discussion

t(9;22), which has diagnostic importance in CML, is detected as a variant at a rate of 5%–10% [9]. Although the FISH test is stated to be a suitable method for the detection of vPh, it is reported in the literature that vPh detection rates are evaluated according to the results of conventional cytogenetic analysis [1, 4, 6, 10]. In the current study, the vPh detection rate based only on conventional cytogenetic analysis was 3.03%, which is consistent with the literature. Conventional cytogenetic analysis is the gold standard for identifying the third or additional chromosomes involved in variant t(9;22). However, as noted by Marzocchi et al. [9], chromosomal abnormalities can be submicroscopic, and therefore the translocation may be masked and revealed only by FISH or molecular analysis. Also, chromosomes cannot always be obtained as a result of conventional cytogenetic studies. For this reason, we aimed to investigate the prognostic effects of nonclassical signal patterns during patient follow-up. In the current study, the rate of cases in which signals other than the classical signal pattern were detected by FISH was 21.21%. This rate is quite high, and aberrant signal patterns should be considered if chromosomes cannot be obtained in conventional cytogenetic studies during patient follow-up. An example of this situation is case #5, whose FISH signal pattern was 1F2O2G at the time of diagnosis and whose chromosomes could not be obtained. At 1 year after the diagnosis, in the study performed with the control bone marrow, the karyotype of the case was reported as 46,XY,der(3)t(3;9;22)(p25;q22;q11.2)t(9;22)(q34;q11.2),der(9)t(3;9;22),der(22)t(9;22)t(3;9;22)[9]/46,XY[12]. It was shown by molecular methods that this case did not achieve MMR.

The higher prevalence of nonclassical FISH signal patterns (21.21%) observed in our study, compared with the 5%–10% range reported in the literature, can primarily be attributed to methodological differences. While previous prevalence estimates were mostly based on conventional cytogenetic analyses, our findings were derived from interphase FISH, which can reveal atypical *BCR::ABL1* signal configurations that may suggest variant or cryptic translocations, particularly in the absence of metaphase chromosomes. In our cohort, conventional cytogenetics failed to yield analyzable metaphases in a considerable number of cases. However, the use of FISH enabled the detection of these nonclassical patterns that could otherwise have remained unrecognized. Moreover, such atypical FISH configurations are often underreported in routine practice, especially when metaphase confirmation is not available. Our systematic evaluation likely contributed to the higher detection rate. These findings underscore the importance of careful interpretation of nonclassical FISH patterns in routine diagnostics, especially in cases where conventional karyotyping is not informative.

There is differing information in the literature about the prognostic effects of vPh. When we evaluated previous studies, we observed that some reports argued that vPh had no prognostic effect [1–3], while others stated that it had a worse prognostic effect [4, 11–13]. Among our case group, vPh was detected in seven cases by conventional cytogenetics and FISH.

**Figure 1:**
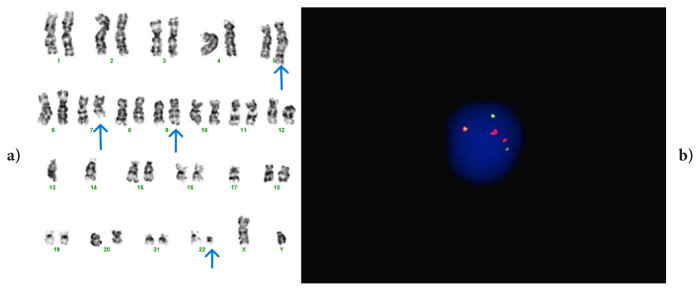
Case #1: t(5;9;22;7)(q31;q34;q11;q31) variant translocation. FISH signal patterns: 2F1O1G and 1F2O2G.

The variant translocation detected in case #1 involved chromosomes 5 and 7. A complex translocation, t(5;9;22;7)(q31;q34;q11;q31), has not been previously reported in the literature (Figures 1a and 1b). As a result of research conducted in the Mitelman database (https://mitelmandatabase.isb-cgc.org/), it was determined that two cases were reported in which the 7q31 region was included in t(9;22), and six cases were reported in which 5q31 was included [14, 15, 16–20]. However, there is no definitive information in the literature about the prognostic effects of these breakpoints, and since the translocation we reported was unique, no clear conclusion can be drawn about its prognostic effect. However, the case we reported (case #1) was diagnosed at the age of 82, received IM treatment, and was CCyR negative at 6 months but MMR positive at 12 months.

**Figure 2:**
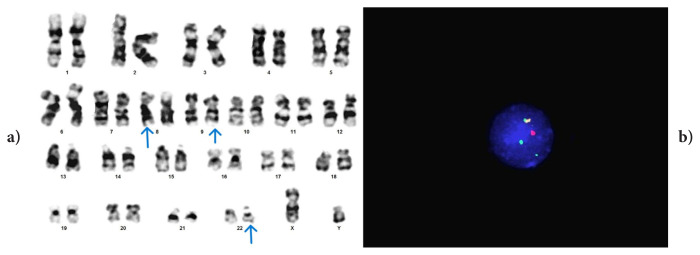
Case #2: t(8;9;22)(p21;q34;q11.2). FISH signal pattern: 1F1O2G.

Only two cases have been reported in the literature where the p21 region of chromosome 8 is included in t(9;22) [21, 22]. One of these cases was a three-way translocation, while the other was a four-way translocation. Our case in which 8p21 was involved in vPh is case #2, and in this case, even after a switch to second-generation TKI, it was determined that CCyR and MMR were not achieved (Figures 2a and 2b). More case reports are needed to elucidate the prognostic impact of this region in vPh.

**Figure 3:**
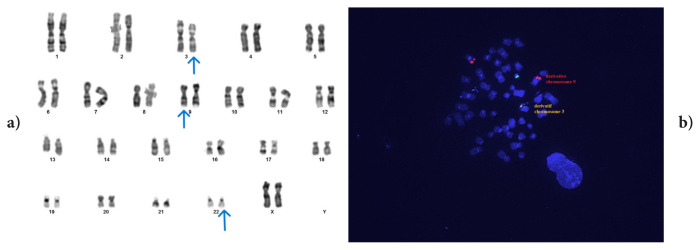
Case #3: der(3)t?(3;22)(p?;q11.2)t(9;22)(q34;q11.2), der(9)t(9;22), der(22)t(9;22)t(3;22). FISH signal pattern: 1F2O1G.

**Figure 4:**
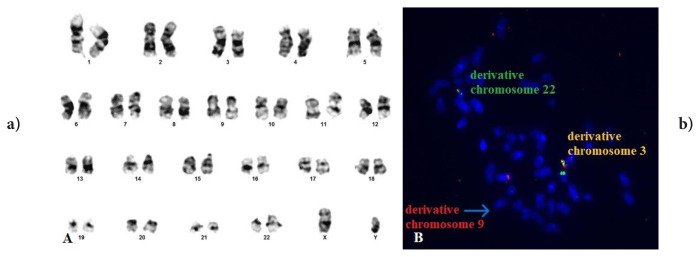
Case #5 (follow-up): t(3;9;22)(p25;q22;q11.2). FISH signal pattern changed to 1F1O2G.

In two cases where we detected variant Ph (cases #3 and #5), we observed that chromosome 3 was included in t(9;22) and that a breakpoint occurred in the short arm in both cases (Figures 3 and 4). The conventional cytogenetic and FISH results of case #3 are shown in Figures 3a and 3b, while those of the other case are shown in Figures 4a and 4b. We determined the breakpoint only in case #5. We determined that four cases with the same anomaly had been reported in the literature. Although individual information on cases with this anomaly could not be accessed, the authors of the studies stated that variant translocations did not have a different prognosis than classical t(9;22) [1, 23]. However, one study reported a case in which t(3;9;22) was detected involving the 3p25 region and stated that IM resistance developed in the case [10]. In case #5, where we detected a breakpoint at 3p25, MMR was not achieved at 12 months despite the initiation of second-generation TKI. In addition, although CCyR positivity was detected at 6 months, the patient has since lost the cytogenetic response.

**Figure 5:**
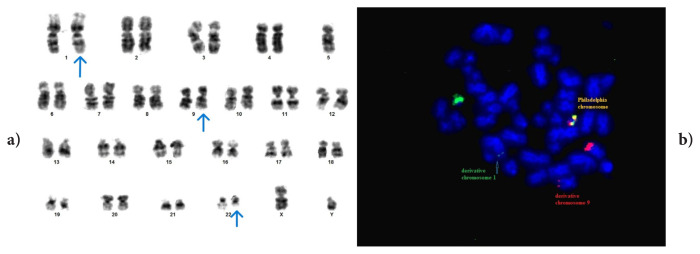
Case #7: Variant translocation involving chromosome 1p36.2. FISH signal pattern: 1F2O1G.

**Figure 6:**
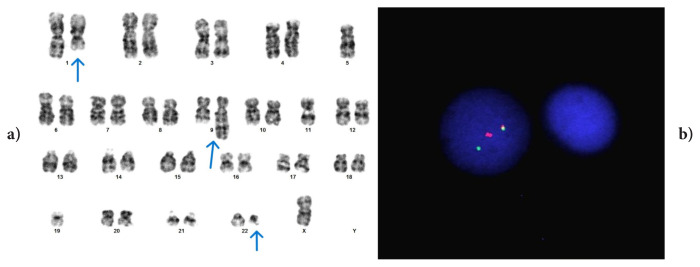
Case #4: 46,XY,t(1;9;22)(q?;q34;q11). FISH signal pattern: 1F1O1G.

In our study, we found that the breakpoint was at p36 in one of the two cases (case #7) where chromosome 1 was involved in the t(9;22) variant (Figures 5a and 5b). Cases in which this region is included in the variant t(9;22) have been reported previously [3, 13, 23–31]. Although clinical information was not available for most of these cases, Zhang et al. [31] reported that a case with a rare transcript, t(1;9;22), showed a better-than-expected clinical response. In the case reported by Lee et al. [27], cytogenetic and molecular responses were also observed. However, in two different cases, it was reported that additional anomalies developed later [25, 30]. In another case, it was reported that no cytogenetic response was obtained [26]. In the case where we detected t(9;22;1), no cytogenetic response was achieved, but a molecular response was obtained after a drug change. In the other case where we detected the variant t(9;22) involving chromosome 1 (case #4), the breakpoint of chromosome 1 was on the long arm, but the exact break region could not be detected (Figures 6a and 6b). In this case, a molecular response was observed after a drug change.

**Figure 7:**
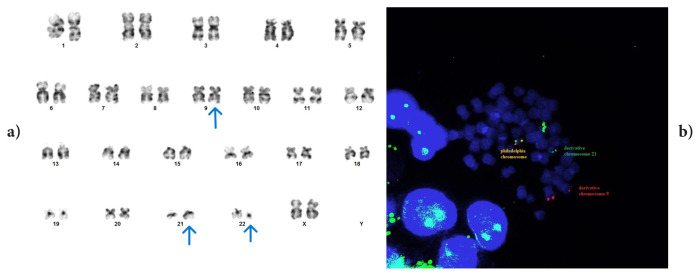
Case #6: 46,XX,t(9;22;21)(q34;q11;q22). FISH signal pattern: 1F2O2G.

Studies reporting variant translocations involving the 21q22 chromosome region in t(9;22) have been published in the literature [1, 3, 4, 25, 32–37]. However, there is no clarity regarding the prognostic effect of this region being involved in the variant translocation. Although there are very limited data in the literature, it is noteworthy that the *RUNX1* gene is localized in this region. Guillaume et al. [34] reported that the third chromosomes included in variant t(9;22)s had no prognostic effect. In the case reported by Bennour et al. [25], the observation of CCyR and MMR at 6 and 12 months, respectively, supports these data. In our reported case #6, although CCyR was not observed at 6 months, MMR was achieved at 12 months without any treatment change (Figures 7a and 7b).

It is evident that a considerable number of case reports are needed to clearly explain the prognostic effects of vPh. Considering the numerous breakpoints reported in the literature, it is understood that a large number of cases are required.

In addition, the effects of deletions observed in variant Ph or classical Ph are also a matter of debate [3]. We investigated the prognostic effect of the nonclassical signal pattern detected by FISH because the frequency of deletions is higher in variant Ph [25], the Ph chromosome can be masked, variant translocations are cryptic, and cytogenetic analyses are not always successful.

When the cases with nonclassical Ph signal patterns detected by FISH were compared with the cases with classical Ph signal patterns and classical t(9;22) detected by cytogenetics in terms of 6- and 12-month treatment responses, drug changes, and overall survival, a difference was observed between the two groups in terms of 6-month treatment responses. These signal patterns indicate *ABL* or *BCR* deletions, or variant Ph. In addition to studies indicating that deletions have a worse prognostic effect [25, 38], there are also studies indicating that they have no different effect from classical t(9;22) [3]. In addition, in our case group with nonclassical FISH signal patterns, 12 cases showed no cytogenetic response at 6 months, while they showed a molecular response at 12 months. The use of second-generation TKIs after 6 months in 7 of 12 cases may have been effective in this change. Although the effect of second-generation TKIs on this condition was found to be statistically insignificant (p = 0.070), it should be reevaluated with a larger number of cases.

Our findings suggest that patients exhibiting nonclassical FISH signal patterns may experience a delayed cytogenetic response compared with those with classical patterns. This observation is clinically significant, as delayed responses could lead to a prolonged period of measurable residual disease, potentially increasing the risk of disease progression. From a therapeutic perspective, such patients may benefit from earlier treatment intensification strategies, such as switching to second-generation TKIs. While current guidelines do not differentiate treatment protocols based on FISH signal variations, our results highlight the importance of individualized cytogenetic monitoring. Future prospective studies are warranted to investigate whether early identification of nonclassical FISH patterns can inform risk-adapted treatment decisions.

In conclusion, our study demonstrated that nonclassical FISH signal patterns were observed in 21.2% of newly diagnosed CML patients, a rate considerably higher than expected based on conventional cytogenetic data alone. This discrepancy highlights the limitations of conventional cytogenetics, particularly in cases where metaphase chromosomes cannot be obtained or where cryptic rearrangements exist. Among the seven patients in whom variant Ph translocations were identified, diverse and previously unreported chromosomal breakpoints were observed, further underscoring the heterogeneity and diagnostic complexity of vPh. Importantly, patients with nonclassical FISH signal patterns showed significantly lower complete cytogenetic response rates at 6 months compared with those with classical patterns, suggesting a possible delay in treatment response. However, a subset of these patients achieved major molecular response at 12 months, often after treatment adjustment, indicating that early cytogenetic delays may be overcome with appropriate therapeutic strategies. These findings emphasize the clinical relevance of careful FISH interpretation in routine diagnostics, especially when conventional cytogenetics is unsuccessful. Incorporating atypical FISH signal patterns into prognostic assessment may allow for more personalized and risk-adapted treatment approaches in CML. Further large-scale prospective studies are needed to clarify the prognostic significance of these findings and to define their role in guiding therapeutic decision-making.

**Supplementary Table:** The FISH signal patterns, karyotypes, treatment information and treatment responses of the cases.

Case no	Sex/Age	FISH signal patern	Karyotype	Therapy	CCyR (6th month)	CCyR (12th month)	MMR (12th month)
1	M/82	2F1O1G/1F2O2G	46,XY,t(9;22)(q34;q11)[3]/46,XY,t(5;9;22;7)(q31;q34;q11;q31)[2]/46,XY[11]	IM	NR	R	R
2	M/42	1F1O2G	46,XY,t(8;9;22)(p21;q34;q11.2)[4]	IM>2.gnr	NR	NA (Ph+ at 9th month)	NR
3	F/18	1F2O1G	46,XX, der(3)t?(3;22)(p?;q11.2) t(9;22)(q34;q11.2), der(9)t(9;22),der(22)t(9;22)t(3;22)[3]/46,XX[20]	IM	NA	NA	R
4	M/71	1F1O1G	46,XY,t(1;9;22)(q?;q34;q11)[3]	IM>2.gnr	R	R	R
5	M/26	D: 1F2O2G	No metaphase plaque	IM>2.gnr	R	NR	NR
		FU: 1F1O2G (12. month)	46,XY,der(3)t(3;9;22) (p25;q22;q11.2)t(9;22)(q34;q11.2), der(9)t(3;9;22), der(22)t(9;22)t(3;9;22)[9]/46,XY[12]				
6	F/60	1F2O2G	46,XX,t(9;22;21)(q34;q11;q22)[18]	IM	NA	NA	R
7	M/47	1F2O1G	46,XY,t(9;22;1)(q34;q11;p36.2?)[5]	IM>2.gnr	NR	NA (Ph+ at 9th month)	R
8	F/51	1F2O1G	46,XX,t(9;22)(q34;q11)[17]	IM>2.gnr	NR	NA	R
9	M/43	1F1O	46,XY,t(9;22)(q34;q11)[10]	IM	NR	NA (Ph+ at 18th Month)	R
10	F/87	1F1O/1F1O1G	46,XX,t(9;22)(q34;q11)[5]	IM	NA	NA	NR
11	M/81	1F1O1G	46,XY,t(9;22)(q34;q11)[16]/46,XY[6]	IM>2.gnr	NA	NA	NR
12	M/62	2F1O1G/1F2O2G	46,XX,t(9;22)(q34;q11)[8]	IM	NA	R	R
13	M/72	1F2O2G	46,XY,t(9;22)(q34;q11)[5]	IM	NA	NA	R
14	M/59	1F1O2G	46,XY,t(9;22)(q34;q11)[7]	IM	R	NA	R
15	F/80	1F1O1G	46,XX,t(9;22)(q34;q11)[4]/46,XX[3]	IM>2.gnr	NA	NA	NR
16	F/58	1F1O2G	46,XY,t(9;22)(q34;q11)[5]	IM>2.gnr	NA	NA	R
17	F/32	1F2O2G	46,XX,t(9;22)(q34;q11)inc[9]	IM>2.gnr	NA	NA	R
18	M/23	1F1O1G	46,XY,t(9;22)(q34;q11)[22]	IM>2.gnr	R	R	NR
19	F/63	1F1O1G	46,XX,t(9;22)(q34;q11)[6]	IM>2.gnr	NR	NA	NR
20	F/36	1F1O2G	46,XX,t(9;22)(q34;q11)[5]	IM>2.gnr	R	NA	R
21	F/60	1F1O2G	46,XX,t(9;22)(q34;q11)[16]	IM	R	NA	R
22	M/21	1F2O2G	46,XY,t(9;22)(q34;q11)[18]	IM	R	NA	R
23	M/39	1F2O2G/2F1O1G/2F2O2G	No metaphase plaque	IM>2.gnr	NA	NA (Ph+ at 9th month)	R
24	M/54	2F1O1G	No metaphase plaque	IM	NA	NA	NR
25	M/84	2F1O1G/1F2O2G	No metaphase plaque	IM	NR	NA	R
26	M/43	1F2O2G	No metaphase plaque	IM>2.gnr	NR	R	NR
27	F/92	1F1O1G	No metaphase plaque	IM	NA	NA	R
28	M/45	2F1O1G/1F1O1G	No metaphase plaque	IM>2.gnr	NR	NA (Ph− at 9th month)	R
29	F/49	1F2O1G	No metaphase plaque	IM	NR	NA	R
30	F/71	1F2O1G	No metaphase plaque	IM	NA	NA	NR
31	M/58	1F1O1G	No metaphase plaque	IM>2.gnr	R	NA (Ph− at 18th month)	R
32	M/69	1F1O1G/1F1O2G	No metaphase plaque	IM	R	NA	R
33	M/49	1F1O2G	No metaphase plaque	IM	R	NA	R
34	M/36	1F2O1G	No metaphase plaque	IM>2.gnr	NR	NA	R
35	M/56	1F1O1G/1F1O2G	No metaphase plaque	IM>2.gnr	R	NA (Ph− at 18th month)	R
36	M/44	1F101G	No metaphase plaque	IM>2.gnr	NR	NA	NR
37	M/31	1F2O2G	No metaphase plaque	IM	NR	NA	R
38	F/60	1F1O1G	No metaphase plaque	IM>2.gnr	NA	NA	R
39	M/54	1F1O1G	No metaphase plaque	IM>2.gnr	NR	NA	NR
40	M/40	2F1O1G/1F2O2G	No metaphase plaque	IM>2.gnr	R	R	R
41	F/77	1F1O1G	No metaphase plaque	IM>2.gnr	R	NA (Ph+ at 18th month)	R
42	M/37	1F2O2G/2F1O1G	No metaphase plaque	IM>2.gnr	NA	NA	NR
43	M/90	1F2O1G/1F2O2G/2F1O1G	No metaphase plaque	IM	NA	NA	NR
44	F/46	1F1O1G	No metaphase plaque	IM>2.gnr	NR	NA	R
45	M/38	1F2O2G	No metaphase plaque	IM	R	NA	R
46	M/85	1F2O2G	No metaphase plaque	IM	NA	NA	R
47	F/87	1F1O1G	No metaphase plaque	IM>2.gnr	NR	NA	NR
48	M/80	2F2O1G	No metaphase plaque	IM>2.gnr	NA	NA	R
49	F/71	1F2O2G	No metaphase plaque	IM	NA	R	NR

F, Female; M, Male; IM: Imatinib mesylate; CCyR, Complete cytogenetic response; MMR, Major molecular response; R, Response positive; NR, No response; NA, Not available; D, Diagnosed; FU, Following up; gnr, Generation; Ph−, t(9;22) negatif; Ph+, t(9;22) Positive

## Figures and Tables

**Table:** Demographic and clinical information of the cases.

	Nonclassic t(9;22) (n = 49)	Classic t(9;22) (n = 73)	p value
Median age (range)	71.33 (48–84)	61.41 (31–87).	0.237
Female/male	21(42.9%)/28(57.1%)	35(47.9)/38(52.1%)	0.713
6th month CCyR (R/NR)	15(45.5%)/18(54.5%)	63(87.5%)/9(12.5%)	**<0.001**
12th month MMR (R/NR)	37(75.5%)/12(24.5%)	58(79.5%)/15(20.5%)	0.771
2.nd generation TKI (+/−)	28(57.1%)/21(42.9%)	28(38.9%)/44(61.1%)	0.073
Survival (alive/ex)	13(27.1%)/35(72.9%)	22(30.6%)/50(69.4%)	0.838
